# Combined dandelion extract and all-trans retinoic acid induces cytotoxicity in human breast cancer cells

**DOI:** 10.1038/s41598-023-42177-z

**Published:** 2023-09-12

**Authors:** Hamed Rezaie, Reza Alipanah-Moghadam, Farhad Jeddi, Cain C. T. Clark, Vahideh Aghamohammadi, Ali Nemati

**Affiliations:** 1 Department of Clinical Biochemistry, School of Medicine, Ardabil University of Medical Sciences, Ardabil, Iran; 2 Department of Genetics and Pathology, School of Medicine, Ardabil University of Medical Sciences, Ardabil, Iran; 3 Centre for Intelligent Healthcare, Coventry University, Coventry, CV1 5FB UK; 4 Department of Nutrition, Khalkhal University of Medical Sciences, Khalkhal, Iran

**Keywords:** Cancer, Diseases, Medical research, Molecular medicine, Oncology

## Abstract

Breast cancer is one of the most prevalent and deadly cancers among women worldwide. Recently, natural compounds have been widely used for the treatment of breast cancer. Present study evaluated antiproliferative and anti-metastasis activities of two natural compounds of dandelion and all-trans-retinoic acid (ATRA) in human MCF-7 and MDA-MB231 breast cancer cells. We also evaluated the expression of MMP-2, MMP-9, IL-1β, p53, NM23 and KAI1 genes. Data showed a clear additive cytotoxic effect in concentrations of 40 μM ATRA with 1.5 and 4 mg/ml of dandelion extract in MCF-7 and MDA-MB231 cells, respectively. In both cell lines, compared with the untreated cells, the expression levels of MMP-9 and IL-1β were significantly decreased while p53 and KAI1 expression levels were increased. Besides, MMP-2 and NM23 had different expressions in the two studied cell lines. In conclusion, dandelion/ATRA co-treatment, in addition to having strong cytotoxic effects, has putative effects on the expression of anti-metastatic genes in both breast cancer cells.

## Introduction

Breast cancer is one of the most prevalent and deadly cancers among women worldwide, in both developed and developing countries^1^. The incidence of breast cancer patients is predicted to reach 3,059,829 people by 2040^2^, and every year, 2,300,000 new cases are diagnosed, with approximately 700,000 deaths yearly, accounting for nearly 6.9% of all cancer deaths^3^. The prevalence of breast cancer varies in different regions of the world, based on race and nationality. Various factors, such as genetics, age, diet, obesity, physical activity, environmental carcinogens, and sex hormones are involved in the etiology of this cancer^4,5^. Breast cancer is characterized by the rapid proliferation of cancer cells and its invasion into healthy tissues, which can reduce patients' survivability^4^. Metastasis is the main leading cause of mortality in patients suffering from this cancer; thus, one of the main defences against breast cancer is the controlling of cancer cells proliferation and prevention of its tissue invasion to identify novel targets for treatment^6,7^. Various chemical drugs have been developed for breast cancer treatment, which have severe side effects, such as cardiac toxicity, nephrotoxicity, and neurotoxicity as well as drug resistance^8–11^. Recently, the use of natural health products (NHPs), as well as plant extracts, as therapeutic agents to ameliorate a wide range of diseases, including cancer, has been considered. Moreover, 60% of the global population is habituated to the consumption of vegetables, fruits, herbs, and vitamins to prevent and/or remedy non-communicable diseases^12,13^. Indeed, natural compounds, in addition to having very strong therapeutic effects, have far fewer side effects compared to chemical anticancer drugs^14,15^. All-trans retinoic acid (ATRA), a derivative of vitamin A, is known as a strong anticancer agent causing growth inhibition, differentiation and apoptosis induction in several human cancers^16^. Moreover, several studies have identified an additive cytotoxic effect of ATRA combined with other natural compounds, such as caffeic acid, genistein, and curcumin, against various cancer cells^17–19^.

*Taraxacum officinale* (Dandelion), belonging to Asteraceae family, has received a lot of attention in recent years. All dandelion organs, i.e. the roots, leaves, and flowers contain significant amounts of flavonoid compounds and l-chicoric acid, which have potent antioxidant, anti-inflammatory, and anti-cancer activities^20,21^. Dandelion is used as traditional folk medicine throughout Asia, Europe and North America to improve outcomes in several diseases^22^. Empirical studies have shown that dandelion extract can inhibit the proliferation of cancer cells and induce death in them^13,23,24^; and has strong anti-metastasis effects on cancer cells such as glioblastoma cells^25^. In addition to breast cancer cells, it has been shown that dandelion potently inhibit proliferation and migration of other cancer cells such as gastric cancer cells^26^. Several studies have also shown that polyphenolic compounds in plant extracts can apply potential anticancer effects in combination with retinoic acid^27–29^. Therefore, this study was designed to evaluate the cytotoxic effect of dandelion/ATRA combination. To achieve this goal, we used MCF-7 and MDA-MB231 well-established breast cancer cell lines which have different phenotypic/genotypic features such as progesterone and estrogen receptor status, different aspects of epithelial or mesenchymal type, multidrug resistance and invasiveness^30,31^. Additionally, we aimed to evaluate the expression level of IL-1β, as a gene that plays a role in cell proliferation, in human breast cancer cells. Thus, along with IL-1β, its downstream genes, MMP-2 and MMP-9, expressions were assessed. To evaluate the anti-metastasis effects of dandelion/ATRA co-treatment, the expression of p53, NM23, and KAI1 genes, which are known as tumor metastasis suppressors, were measured.

## Results

### Effect of dandelion extract, ATRA, or dandelion/ATRA co-treatment on cell viability

**Figure 1:**
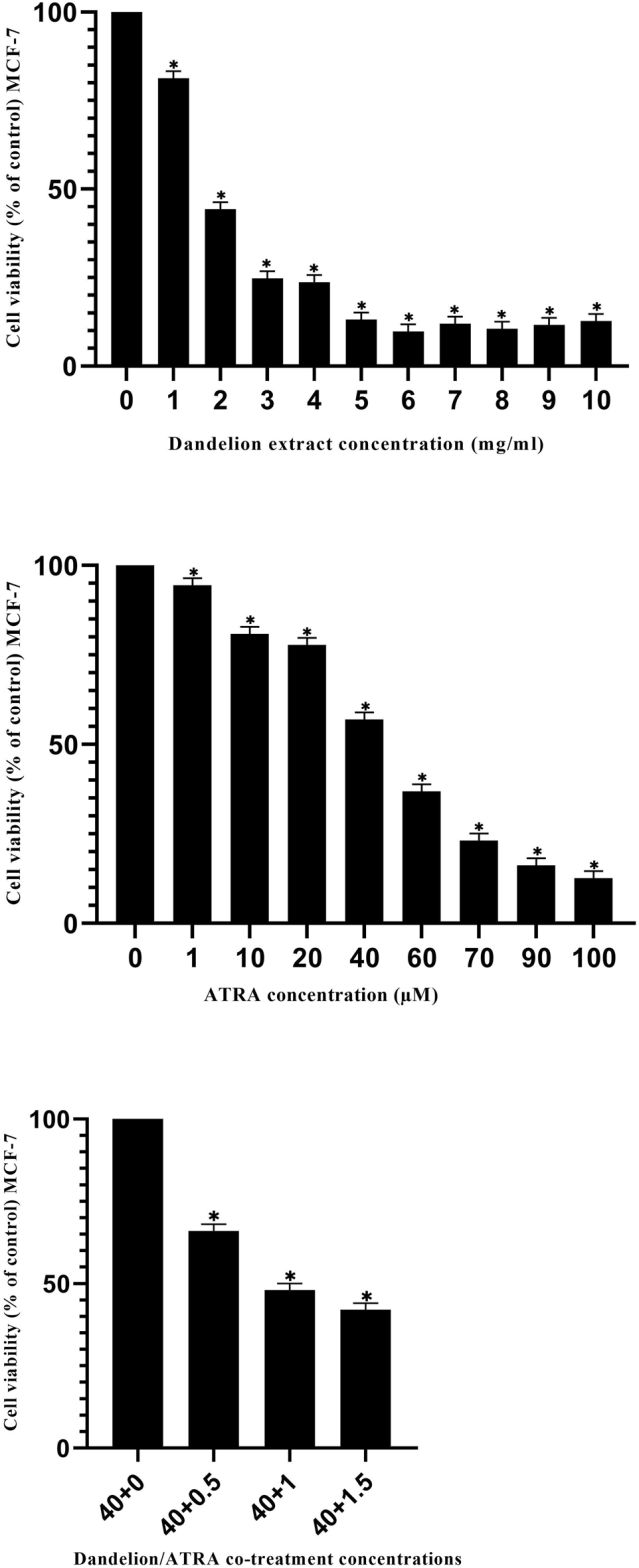
The effect of dandelion or ATRA and dandelion/ATRA co-treatment on MCF‑7 cell viability. Viability was assessed with the MTT method after the exposure to the increasing concentrations of components for 48 h. The data are the mean ± SD values of triplicate individual determinations. The lowest cytotoxic concentrations of dandelion extract and ATRA were observed in concentrations of 1 mg/ml and 1 µM, respectively. Also, the highest cytotoxic concentrations of dandelion extract and ATRA on cell viability occurred in concentrations of 10 mg/ml and 100 µM, respectively. To evaluate the effect of dandelion/ATRA co-treatment on cell viability, the concentration of ATRA was fixed at 40 µM and the concentration of dandelion was considered variable between 0 and 1.5 mg/ml (*P < 0.05).

MCF-7 cells were cultured in DMEM medium with 10% FBS and seeded in 96-well plates, after 24 h and when cell density reached 80–90%, the cells were treated as triplicates with previously mentioned concentrations of dandelion extract, ATRA, or the combination of dandelion extract and ATRA. After 48 h of treatment, cytoviability was assessed by the MTT test. Exposure of MCF-7 cells to all three compounds inhibited cell survival in a dose-dependent manner. The lowest cytotoxic concentrations of dandelion extract and ATRA on cell viability occurred in concentrations of 1 mg/ml and 1 µM, respectively, as well as a combination of these concentrations (Fig. 1). Also, the highest cytotoxic concentrations of dandelion extract and ATRA on cell viability occurred in concentrations of 10 mg/ml and 100 µM, respectively, as well as a combination of these concentrations. According to the dose–response curve, the IC50 values for dandelion extract and ATRA were 1.69 mg/ml and 48 μM, respectively. The concentrations of dandelion extract and ATRA used for the final treatment were considered at 1.5 mg/ml and 40 μM, respectively (Fig. 1).

**Figure 2:**
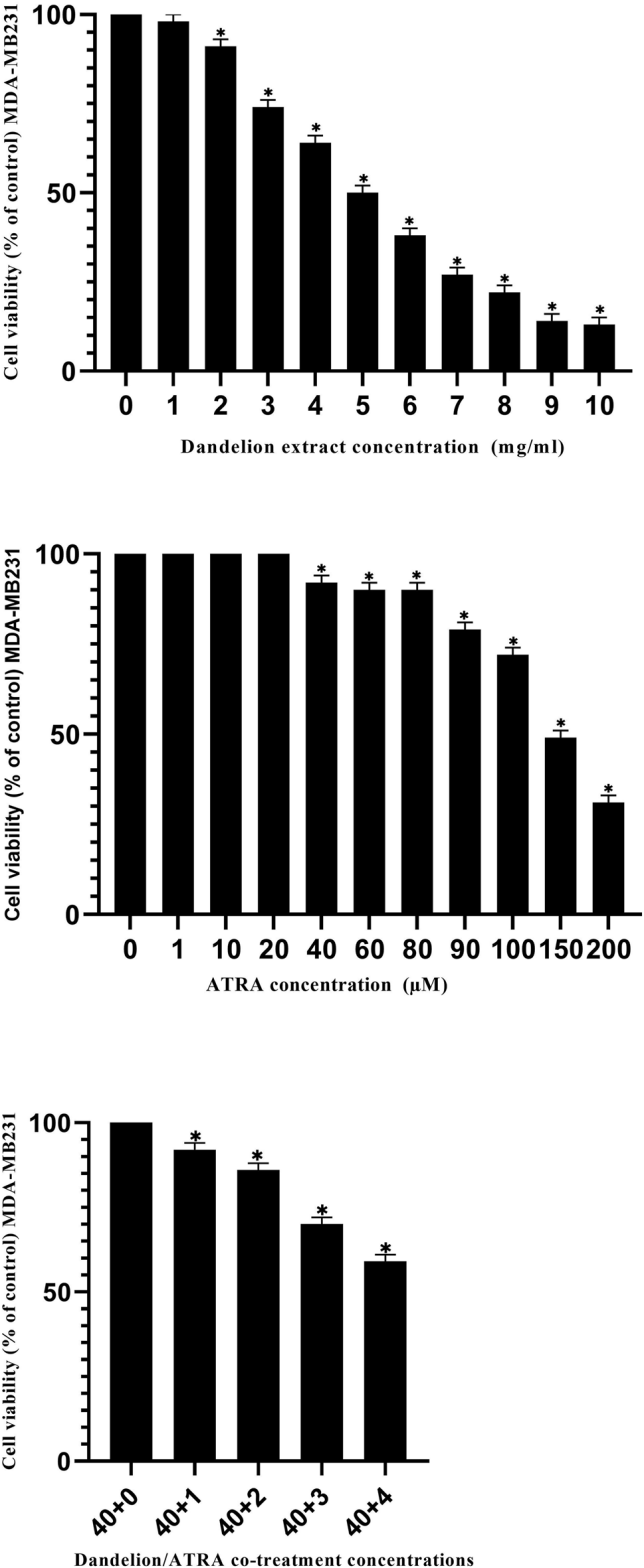
The effect of dandelion or ATRA and dandelion/ATRA co-treatment on MDA-MB231 cell viability. Viability was assessed with the MTT method after the exposure to the increasing concentrations of components for 48 h. The data are the mean ± SD values of triplicate individual determinations. The minimum cytotoxic effects of dandelion and ATRA were seen in 1 mg/ml and 1 µM concentrations, respectively. Moreover, the maximum cytotoxic effects of dandelion and ATRA were observed in 10 mg/ml and 200 µM concentrations, respectively. To estimate the effect of dandelion/ATRA co-treatment on cell viability, the concentration of dandelion was considered variable between 0 to 4 mg/ml and the ATRA concentration was fixed at 40 µM. (*P < 0.05).

Similarly, the effect of dandelion extract, ATRA, or the combination of dandelion extract and ATRA, on cell viability of MDA-MB231 cells was studied. Based on the results of the MTT test, the lowest cytotoxic concentrations of dandelion extract and ATRA on cell viability occurred in concentrations of 1 mg/ml and 1 µM, respectively, as well as a combination of these concentrations (Fig. 2). Furthermore, the highest cytotoxic concentrations of dandelion extract and ATRA on cell viability occurred in concentrations of 10 mg/ml and 200 µM, respectively, as well as a combination of these concentrations. Using Sigma Plot software, the IC50 values for dandelion extract and ATRA were calculated at 5.9 mg/ml and 149 μM, respectively. The concentrations of 4 mg/ml dandelion extract and 40 μM ATRA were used to evaluate their individual or combined effects on MDA-MB231 cells (Fig. 2).

### Treatment concentrations for gene expression

In MCF-7 and MDA-MB231 cells, gene expression were assessed in cells treated with the concentrations of 1.5 and 4 mg/ml of dandelion extract, respectively, and constant concentration 40 µM of ATRA. To determine gene expression in dandelion/ATRA co-treated cells, the concentrations of 1.5 mg/ml dandelion with 40 µM ATRA and 4 mg/ml dandelion with 40 µM ATRA were used in MCF-7 and MDA-MB231 cells, respectively.

### Effect of dandelion extract or ATRA and dandelion/ATRA co-treatment on MMP-2 expression

**Figure 3:**
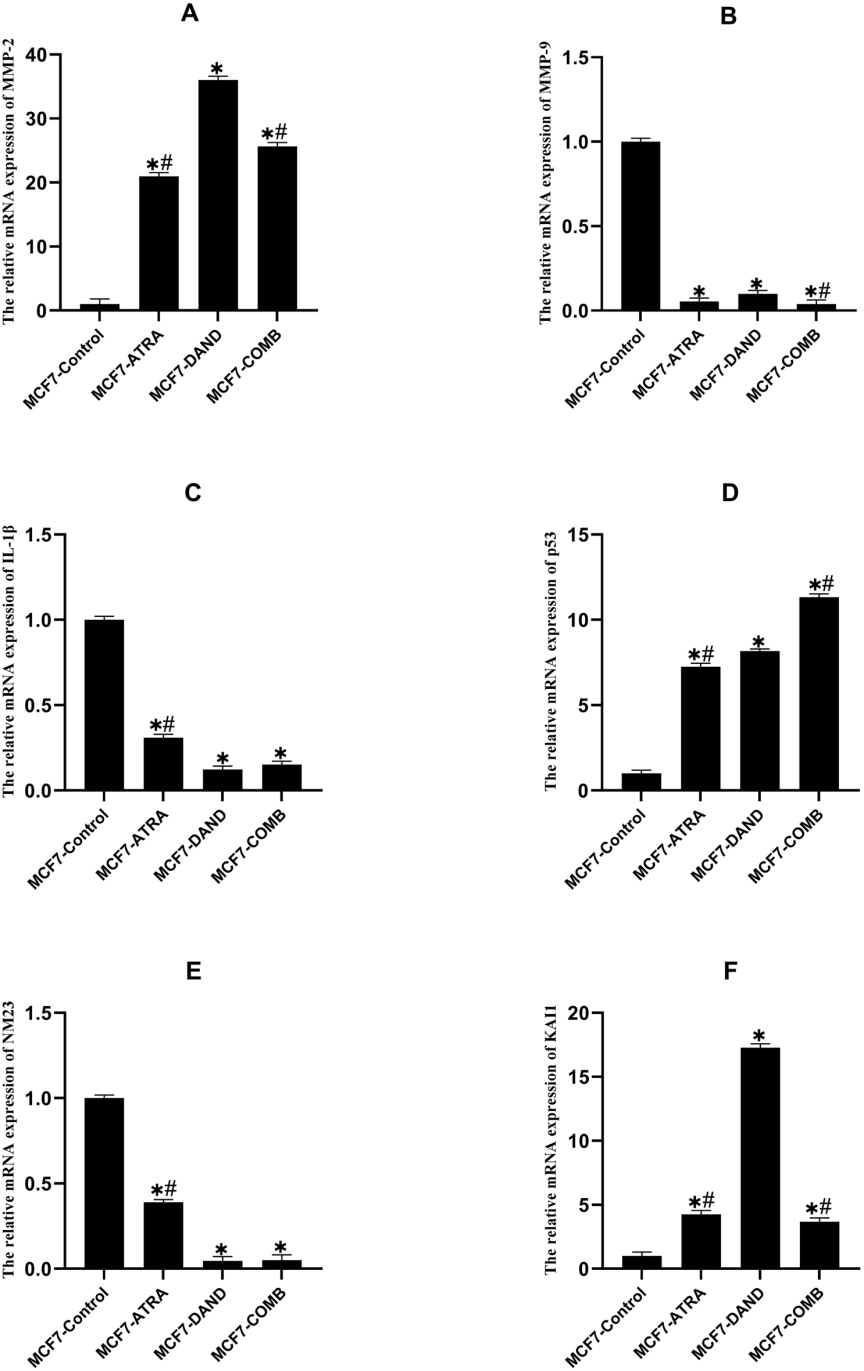
The Effect of dandelion or ATRA and dandelion/ATRA co-treatment on gene expression in MCF-7 breast cancer cells. (**A**–**F**) Real time PCR method was done to calculate the expressions of MMP-2, MMP-9, IL-1β, p53, NM23, and KAI1. The data is represented as the mean ± SD of 3 independent determinations. (*P < 0.05).

**Figure 4:**
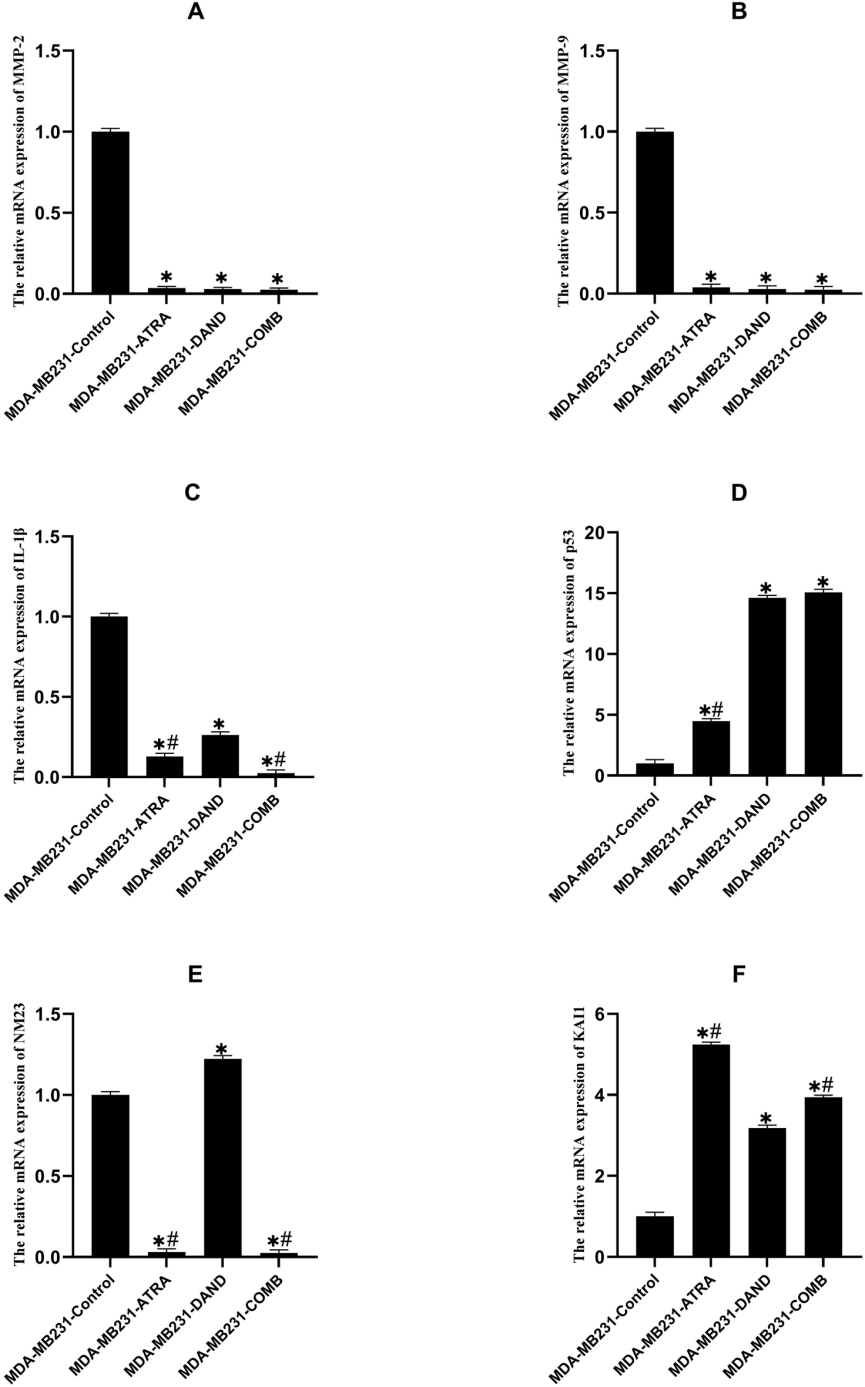
The Effect of dandelion or ATRA and dandelion/ATRA co-treatment on gene expression in MDA-MB231 breast cancer cells. (**A**–**F**) The mRNA expressions of MMP-2, MMP-9, IL-1β, p53, NM23, and KAI1 were carried out by quantitative real time PCR. The data are the mean ± SD values of triplicate individual determinations. (*P < 0.05).

In the MCF-7 cells, in all treated groups, MMP-2 expression level was significantly higher compared to the untreated control group (p˂0.05) (Fig. 3A). The highest MMP-2 mRNA expression was found in the dandelion extract-treated group, compared to the other groups (p < 0.05). Also, the MMP-2 expression level in the combined group was higher than the ATRA-treated group (p < 0.05). However, in the MDA-MB231 cells, the MMP-2 expression was shown to decrease significantly in all the treated groups, unlike in the untreated control group (p˂0.05). There is no significant difference in the MMP-2 mRNA expression among the treatment groups (p > 0.05) (Fig. 4A).

### Effect of dandelion extract, ATRA, or a combination of dandelion extract and ATRA, on MMP-9 expression

Our results indicated that MMP-9 expression in both MCF-7 and MDA-MB231 cells had a significant decrease in the treated groups compared to the untreated control group (p < 0.05). However, the expression of MMP-9 declined in the combination group in MCF-7 cells compared to the dandelion group (p > 0.05), but its association with the ATRA group was not significant (p > 0.05) (Fig. 3B). Likewise, in the MDA-MB231 cell line, the MMP-9 mRNA expression level was not significantly different between the treatment groups (p > 0.05) (Fig. 4B).

### Effect of dandelion extract, ATRA, or a combination of dandelion extract and ATRA on IL-1β expression

Based on our results, IL-1β expression in both cell lines were decreased in the treatment groups with dandelion extract, ATRA, or their combination, compared to the untreated control group (p < 0.05). Interestingly, the reduction in IL-1β expression level in MCF-7 cells in dandelion and combined groups was much greater than in the ATRA group (p < 0.05). However, dandelion elicited a further reduction in expression of IL-1β compared to the combined group, although this was not significant (p > 0.05) (Fig. 3C). IL-1β gene expression in the MDA-MB231 cell line was significantly reduced in the combined group compared to the ATRA and dandelion groups, moreover, ATRA elicited a further reduction in expression of IL-1β compared to the dandelion group (p < 0.05) (Fig. 4C).

### Effect of dandelion extract, ATRA, or a combination of dandelion extract and ATRA on p53 expression

Based on analysis of variance, p53 mRNA expression significantly increased in MCF-7 cells after incubation with dandelion extract, ATRA, or a combination of both, compared to the untreated control group (p < 0.05). Surprisingly, a significant increase in the p53 gene expression was detected in the combined group compared to both the dandelion and ATRA groups (p < 0.05). There were significant changes in the p53 mRNA levels between dandelion and ATRA groups (p < 0.05) (Fig. 3D). In MDA-MB231 cells, the rate of p53 mRNA expression levels was significantly increased in all treatment groups compared to the untreated control group (p < 0.05), however, the p53 expression level in dandelion and combined groups was similarly higher (Fig. 4D).

### Effect of dandelion extract, ATRA, or a combination of dandelion extract and ATRA on NM23 expression

Our results showed that NM23 expression in MCF-7 cells, after 48 h of exposure to dandelion extract, ATRA, or a combination of both, decreased significantly compared to the compared to the untreated control group control group (p < 0.05). In addition, the dandelion and dandelion/ATRA co-treatment significantly reduced NM23 expression in MCF-7 cells compared to the ATRA group (p < 0.05). However, there was no significant difference in the NM23 mRNA levels between dandelion and combined groups (p > 0.05) (Fig. 3E). NM23 expression in MDA-MB213 cells decreased significantly in ATRA and dandelion/ATRA co-treatment, as compared to the untreated control group (p < 0.05). Additionally, dandelion significantly increased NM23 expression in MDA-MB231 cells compared to other groups (p < 0.05) (Fig. 4E).

### Effect of dandelion extract, ATRA, or a combination of dandelion extract and ATRA on KAI1 expression

The results of the present study showed that the expression of KAI1 was increased in MDA-MB231 and MCF-7 cells after 48 h in treated groups compared to the untreated control group (p < 0.05). Furthermore, a significant increase in KAI1 expression was detected in MCF-7 cells after treatment by dandelion, compared to the ATRA and combined groups (p < 0.05). In MCF-7 cells no significant correlation was observed between ATRA and combined groups (p > 0.05) (Fig. 3F). In MDA-MB231 cells, the ATRA group significantly increased KAI1 expression, as compared to the dandelion and dandelion/ATRA co-treatment (p < 0.05). In addition, the combination of ATRA with dandelion significantly increased KAI1 expression in MDA-MB231 cells compared to the dandelion group (p˂0.05) (Fig. 4F).

As confirmed in our study, the combination of dandelion and ATRA elicited cell cytotoxicity following overexpression of p53 in both MCF-7 and MDA-MB231 breast cancer cells. The p53 gene, as a cell guard, is critically involved in programmed cell death, cell cycle regulation, and DNA repair, and, in addition to stimulating the apoptotic pathway, the p53 protein suppresses the expression of the anti-apoptotic Bcl-2 and Bcl-XL genes in its normal function^32^. Choi et al. studied the proapoptotic effect of ethanol extracts acquired from the dandelion flower on human ovarian cancer SK-OV-3 cells and stated that apoptosis induction by dandelion involved p53 activation, bax upregulation, and downregulation of Bcl-2^33^. In the study by Chen et al. administration of dandelion leaf polysaccharide in H22 tumor-bearing mice directly induced tumor cell apoptosis via upregulation of p53 and pro-apoptotic protein Bax expression^34^. George and Abrahamse used Rubus fairholmianus derivatives against MCF-7 breast cancer cells and reported that overexpression of p53 in MCF-7 cells treated with these derivatives induced apoptosis in MCF-7 cells^35^. Furthermore, Motadi et al. indicated the anticancer effects of Tulbaghia violacea extract in several cancer cell lines, such as MCF-7 and MDA-MB231 following p53 overexpression. The authors concluded that the p53-dependent pathway may be responsible for cell death^36^. In addition, Won et al., using Sanggenol L, as a natural flavonoid, showed apoptosis in human prostate cancer cells can be directed via suppression of PI3K/Akt/mTOR signaling and cell cycle arrest via activation of p53^37^. Previous studies have also evaluated the combination of ATRA with other anti-cancer agents. Wang et al. indicated that ATRA and decitabine in combination synergistically induce apoptosis in higher-risk myelodysplastic syndromes and elderly acute myeloid leukaemia cells^38^. Another study also showed that there was an additive effect in leukemic cells when treated with a combination of ATRA and epigallocatechin‑3‑gallate (EGCG), as active polyphenolic compounds of green tea. The authors concluded that the cytotoxic effects of EGCG were enhanced by ATRA^39^. In this study, we present a novel finding that shows an additive cytotoxic effect of the combination of dandelion with ATRA on breast cancer cells followed by overexpression of p53, which indicates the possible role of p53-related pathways in this process.

Along with the increase in the expression of p53, we observed a marked decrease in the expression of IL-1β in MCF-7 and MDA-MB231 cells treated with dandelion extract, ATRA, or combination of both. It has been shown that IL-1β level is associated with the progression and stage of breast cancer^40,41^. In this regard, it has been reported that IL-1β progresses tumorigenesis through attenuation of p53 protein expression and promotion of the growth of different types of cancer cells by nuclear factor kappa B (NF-κB) activation^42–44^. Suppression of IL-1β expression decrease tumor growth and inhibit perfusion of tumor cells from the primary location into circulation^45–48^. Therefore, regarding central role of IL-1β in growth and proliferation of breast cancer cells, our findings may be defined as a part of the mechanism of cytotoxicity by dandelion extract, ATRA, or the combination of both, on breast cancer cells, MCF-7, and MDA-MB231. Several studies indicated the anti-inflammatory effects of dandelion extract^49,50^. In the study by Liu et al. dandelion extract significantly decreased the expression of IL-1β, IL-6, and TNF-α, and reduced the LPS-induced inflammatory response^51^. Studies have indicated that some of compounds in dandelion extract such as phenolic acids, flavonoids, triterpenes and sterols have potential therapeutic effects in inflammation^51–55^. Moreover, anti-inflammatory effect of ATRA has been demonstrated in previous studies^56,57^. However, Guo et al. reported that ATRA may stimulate the expression of IL-1β by activating NF-κB signalling and caspase-1 in macrophages^58^. Furthermore, a study indicated that the anti-proliferative effect of ATRA is associated with selective stimulation of IL-1β, a cytokine that directly prevents growth of lung cancer cells^59^.

Based on our results, dandelion, alone or combined with ATRA, reduced MMP-2 and MMP-9 expressions in MDA-MB231 cells, while also reducing MMP-9 expression in MCF-7 cells, and increasing MMP-2 expression in these cells. Despite that different genes contribute to the control of metastasis, the role of matrix metalloproteinases (MMPs) are more pronounced. MMPs are a group of zinc-containing enzymes that play a key role in various processes, such as inflammation, angiogenesis, tissue regeneration, and invasion of cancer cells into other tissues. Among the MMPs, MMP-2 and 9 play the most prominent role in metastasis. Several studies have also shown that MMP-2 and MMP-9 play a key role in the invasion of cancer cells, and their increased expression is directly related to the malignancy and aggressiveness of cancer cells^60,61^. Furthermore, it has been shown that the attenuation of MMP-2 and 9 expressions can greatly reduce the invasiveness of cancer cells. Moreover, it has been found that MMP-9 is expressed in small amounts by healthy breast tissue, but it notably increases in malignant breast cancer cells as well as in the metastatic stage of breast cancer^62,63^. On the other hand, MMP-9 expression reduction can inhibit the aggressive malignancy of cancer cells^64^. Moreover, multiple studies emphasized the role of MMP-2 in metastasis in various cancers, including breast cancer^65,66^. Furthermore, other natural products, such as curcumin, quercetin, and polyphenol, inhibit MMP-2 and MMP-9 expressions and tumor metastasis^67^. There are several studies confirming our results regarding MMP-9 expression, such as Liang et al. and Liu et al., who showed that ATRA decreased the expression of MMP-9 in glioma and MDA-MB-231 cells^68,69^. However, consistent with the results obtained in our study in MCF-7 cells, which showed that the expression of MMP-2 was increased in the treatment groups, Malmira et al. showed that colorectal cancer HCT-116 cells treated using ginger extract, significantly increased MMP-2 expression level in a dose-dependent manner^70^. Furthermore, it has been shown that ATRA has an effective role in the regulation of MMP-2 or MMP-9 expression in different cells^71^. Although, there are conflicting reports in the literature regarding the effect of ATRA on the expression of MMP-2 and 9, such that, in some studies, the expression of MMP-2 and MMP-9 was inhibited by ATRA. However, in other studies, the expression of MMP-9 was reduced and the expression of MMP-2 remained unchanged, whilst another study reported that both MMP-2 and MMP-9 expression were elevated^68,72,73^. Consistent with the results obtained in our study in MCF-7 cells, Vu et al. showed that ATRA increases MMP-2 expression and secretion in human myeloid leukemia THP-1 cells^74^. Also, Kim et al., by investigating the effect of ATRA on the expression of MMP-2 and 9 in human dental pulp cells (HDPCs), showed that ATRA decreases the expression of MMP-2 in these cells, but has no effect on the expression of MMP-9^75^. The amplification effect of ATRA, in combination with other natural products including curcumin and vitamin D, on cell death and the invasion of cancer cells has also been investigated in various studies^19,76^. Overall, the effect of dandelion, or its combination with ATRA, on the expressions of MMP-2 and MMP-9 in MDA-MB231 and MCF-7 cells should be considered a novel finding that can be used in cancer treatment strategies by conducting further research. Another finding in our study is the significant increase in KAI1 expression in both MCF-7 and MDA-MB231 cells in all treatments consisting of dandelion, ATRA, or the combination of ATRA with dandelion. The KAI1 or CD82 gene is a major tumor metastasis suppressor that primarily inhibits cancer cell motility and invasiveness in a wide variety of solid malignant tumors, such as prostate, stomach, colon, hepatocarcinoma, thyroid, pancreas, and breast cancers. KAI1/CD82 may be regarded as a main prognostic biomarker in anticipating metastatic development and maybe a hopeful candidate for therapeutic purposes in cancers^77,78^. The expression of KAI1 is abundant in normal breast tissue, but its expression is reduced in primary tumors, as well as in the metastatic stage of breast cancer^79^. KAI1 inhibits cancer cell migration and adhesion, in addition to apoptosis, by preventing integrin-mediated signalling^80^. Moreover, KAI1 upregulates tissue inhibitor of metalloproteinase 1(TIMP1), which causes the inactivation of MMP-9. Restored KAI1 expression is significantly associated with loss of different cancer cell proliferation, such as ovary, stomach, pancreas, and breast^77,79^. Thus, the increase in the expression of KAI1 in our treatment groups can indicate the effect of dandelion and ATRA on reducing the aggressiveness of breast cancer cells. Also, interestingly, our results showed a reducing effect of ATRA, or its combination with dandelion, on NM23 expression in both MCF-7 and MDA-MB231 cells. NM23 is considered as a potential anti-invasive and tumor-metastasis suppressor, moreover, it has been shown that low NM23 expression may be associated with a poor prognosis in breast cancer^81^. However, in our study, elevated expression in NM23 was observed only in the dandelion-treated group in MDMB-231 cells, as compared to the control group, which should be studied so to elucidate possible mechanistic actions. Overall, it appears that dandelion, or its combination with ATRA, can be very effective in inducing cell death and controlling the main agents involved in the invasion of breast cancer cells.

The present study highlighted that dandelion extract, in combination with ATRA, in addition to having strong cytotoxic effects on breast cancer cells, also has potential effects on the expression of genes involved in the proliferation and invasion of MCF-7 and MDA-MB231 breast cancer cells.

## Materials and methods

### Materials

MCF-7 and MDA-MB231 human breast cancer cell lines were obtained from the Pasteur Institute of Iran, Tehran. DMEM (Dulbecco's Modified Eagle Medium) (Cat. No: 12800-116), FBS (Fetal Bovine Serum cell culture) (Cat. No: 10270), penicillin/streptomycin (Cat. No: 15140), Glutamax (Cat. No: 25030) and non-essential amino acids (Cat. No: 11140050) were purchased from Thermo Fisher Scientific Inc. (UK). Cell Proliferation Kit (MTT) (Cat. No: M2128), DMSO (Dimethylsulfoxide) (Cat. No: D4540), and ATRA (Cat. No: R2625-50 Mg) were purchased from Sigma-Aldrich, USA. Trypsin (X0930) was purchased from Biowest, France. Dandelion root was prepared from the Namin region of Ardabil province, Iran (Latitude of 38°25′ 28.04″ N, Longitude 48°28′ 41.67″ E). Plant material was collected in accordance with applicable national and international guidelines^82^. Plant specimens were identified by the Research Institute of Forests and Rangelands (RIFR), Tehran, Iran. Voucher specimens were deposited in the Research Institute of Forests and Rangelands (RIFR), Tehran, Iran with voucher Numbers: TARRI 107642.

### Preparation of hydro alcoholic extract of dandelion root

Briefly, the root of the plant was washed with water and dried in air. After complete drying, it was gently powdered by an electric grinder. Then, obtained powder was mixed with 70% ethanol in a ratio of 1:10 w/v. The mixture was then shaken in room temperature for 72 h. The mixture was then filtered using Whatman No. 42 filter paper and dried at 50 °C. The extract obtained were then stored in the dark in a freezer at − 70 °C until use.

### Cell culture

MCF-7 and MDA-MB231 cells were grown and maintained in DMEM supplemented with 10% FBS, which added penicillin/streptomycin (P/S), and kept in a humid atmosphere at 37 °C with 5% CO_2_.

### Tetrazolium (MTT) assay for measuring cellular viability and drug treatment

3-(4, 5)-Dimethyl imidazole (-Zyl)-3, 5-diphenyltetrazolium bromide used for assessment of cell proliferation and cell viability. In this method, the metabolic activity of the cells is determined by reducing the yellow tetrazole to purple formazan in living cells by the action of the mitochondrial succinate dehydrogenase enzyme. MCF-7 and MDA-MB231 cells were cultured in a 96-well plate with a density of 5000 cells per well. After 24 h, MCF-7 cells were exposed to increasing concentrations of dandelion extract (0–10 mg/ml), ATRA (0–100 µM), or the combination of dandelion extract and ATRA (in the same concentration ranges) to determine cell viability. Similarly, MDA-MB231 cells were exposed to increasing concentrations of dandelion extract (0–10 mg/ml), ATRA (0–200 µM) or the combination of dandelion extract and ATRA (in the same concentration ranges) to determine cell viability. Next, plates containing cells were incubated for 48 h at 37 °C in a CO_2_ atmosphere. After 48 h, the medium was removed from the wells and culture medium containing 5 mg/ml of MTT solution was added to each well and cells were incubated for 4 h at 37 °C with 5% CO_2_. DMSO was then added to the wells to dissolve formazan crystals by shaking them for 15 min. The absorbance was measured at 570 nm on an ELISA reader (Synergy HT, USA Biotech, USA). Cell viability at all concentrations was calculated relative to untreated control and as a percentage. It was repeated three times for all groups. Fifty percent inhibitory concentration (IC50) was calculated using Sigma Plot Software version 14. In this study, different concentrations of dandelion and ATRA were evaluated in combined therapy, and based on the Bliss independence equation the results showed that the most effective combined dose was the concentration lower than IC50.^83^.

### Real-time PCR

Briefly, RNA isolation and qPCR studies were conducted according to the MIQE guideline standards^84^. Total RNA was extracted from cultured cells, after 48 h treatment, using Trizol solution as recommended by the manufacturer (Biobasic, Canada). RNA integrity was confirmed by gel electrophoresis on a denaturing 1.5% agarose gel (28S/18S rRNA ratio). Nucleic acid purity and quantity was evaluated on a NanoDrop (Thermo Scientific Nanodrop 2000c, USA) where the A260/A280 & A260/A230 ratios were acquired. RNAs with a ratio of 1.7–2.1 were used for gene expression analysis. Three microgram total RNA was reverse transcribed using SMOBIO Kit (RP1300, Taiwan) in 20 μl volume according to the manufacturer’s procedure. The cDNA was diluted 1:5 in nuclease free water and then real-time PCR was performed by using SYBR Green PCR master mix (Takara TB Green Premix Ex Taq II, RR820Q, Japan). All experiments were conducted on a Real-time PCR Detection System (Ruche light cycler 96, Germany). The PCR reaction was performed in a total volume of 20 μl (10 μl master mix, 1 μl cDNA, 1 µl primer Forward, 1 µl primer Reverse, and 8 μl nuclease free water). The real-time PCR protocol for evaluating each gene expression is provided in the Supplementary Table S1. Oligo Primer Analysis Software (version 7.60) was used for primer designing. Primers and sequencing information are shown in Table 1. For all target and housekeeping genes considered, standard curves were calculated with serial tenfold dilutions of control cDNA to determine amplification *efficiency*. Product specificity was assessed by melting curve analyses and the non-template control (NTC) and genomic DNA (gDNA) samples. Only primer pairs which did not result in more than one melting peak or any sigmoid curve in the NTC and gDNA samples were used for final analyses. Beta-actin (ACTB), as a housekeeping gene, was used as an internal control for normalization. The relative mRNA expression levels were obtained according to the 2^(−ΔΔCt) method^85^. Each measurement was performed in triplicate.

**Table 1:** Primers and sequencing information.

Gene	Gen ID	Primer sequences (5′ to 3′)	Product size (bp)
MMP2	4313	F: GGC GAT GGA TAC CCC TTT GAC	114
		R: TCC CAA GGT CCA TAG CTC	
MMP9	4318	F: CTG GGC AGA TTC CAA ACC TTT	91
		R: GCG GCA AGT CTT CCG AGT AG	
IL-1β	3553	F: CTG GTA CAT CAG CAC CTC TCA	134
		R: GCA CAG GAC TCT CTG GGT ACA	
p53	7157	F: CACAGCACATGACGGAGGTT	70
		R: GCCAGACCATCGCTATCTGA	
NM23	4830	F: TCATGCTCGGGGAGACCAA	126
		R: CGATCTCCTTCTCTGCACTCT	
KAI1	3732	F: GGGAAGAGGACAACAGCCTTT	112
		R: ATGCAGCCCTCCTGGTACAC	
β-actin	60	F: ACAGAGCCTCGCCTTTGC	76
		R: CGCGGCGATATCATCATCCA	

### Supplementary Information

Supplementary Table S1.

## Data Availability

All data and materials are fully presented in the manuscript.

## Abbreviations

ATRA: All-trans retinoic acid
DEPC: Diethyl pyro carbonate
DMSO: Dimethylsulfoxide
FBS: Fetal Bovine Serum
IC50: Fifty percent inhibitory concentration
KAI1 (CD82): Kangai-1 Protein
IL-1β: Interleukin-1beta
MDM2: Murine double minute 2
MMP-2: Matrix Metalloproteinase -2
MMP-9: Matrix Metalloproteinase- 9
NF-κB: Nuclear factor kappa B
NHPs: Natural health products
PBS: Phosphate buffered saline
