# Supplementary material for: Combined dandelion extract and all-trans retinoic acid induces cytotoxicity in human breast cancer cells
Source: Sci Rep. 2023 Sep 12;13:15074. doi: 10.1038/s41598-023-42177-z (PMC10497591; doi:10.1038/s41598-023-42177-z)
Supplement: Supplementary file 1 — Supplementary Table S1. [file 41598_2023_42177_MOESM1_ESM.docx]

Table S1**.** Thermal cycler protocol of real-time PCR for experimented genes

| 1 cycle | 10 min | 95°C | **Preincubation** | |
| --- | --- | --- | --- | --- |
| 35 cycles | 10 sec | 95°C | **Denaturation** | |
|  | 30 Sec | 60°C | MMP-2 | **Annealing** |
|  | 45 Sec |  | MMP-9 |  |
|  | 25 Sec |  | IL-1β |  |
|  | 45 Sec |  | p53 |  |
|  | 30 Sec |  | NM23 |  |
|  | 45 Sec |  | KAI1 |  |
|  | 15 Sec | 72°C | **Extension** | |
